# Azobenzene Based Photo-Responsive Hydrogel: Synthesis, Self-Assembly, and Antimicrobial Activity

**DOI:** 10.3390/gels8070414

**Published:** 2022-07-01

**Authors:** Runmiao Yang, Wei Jin, Chingcheng Huang, Yuhai Liu

**Affiliations:** 1 Department of Material Engineering, Jiangsu University of Technology, Changzhou 213001, China; jinweijsut@163.com (W.J.); lyh@jsut.edu.cn (Y.L.); 2 PARSD Biomedical Material Research Center, Changzhou 213001, China; jcchuang@mail.mcu.edu.tw; 3 Department of Biomedical Engineering, Ming-Chuan University, Taoyuan 333, Taiwan

**Keywords:** azobenzene, hydrogel, quaternary ammonium salt, antimicrobial activity

## Abstract

A new azobenzene-based symmetric amphiphile was synthesized and characterized using ^1^H NMR spectroscopy. Its self-assembly behavior as well as photo-responsive behavior in its solution and gel states were investigated. Such a compound can self-assemble into fiber mesophases in water solvent. After irradiation of the gels with UV light, the *trans* isomer of the compound rapidly photoisomerized to the *cis* isomer, which resulted in a rapid destruction of the gel. High temperature also caused a rapid drop in viscosity. To verify the antimicrobial activity of the hydrogel, live and death assays of human fibroblasts L929 properties were used for in vitro cell viability studies. The compound was converted to the terminal tertiary amine in a quaternary ammonium salt molecule by using hydrochloric acid. This azobenzene quaternary ammonium salt has a relatively better antimicrobial effect biocidal activity that was demonstrated when challenged against Escherichia coli on in vitro conditions.

## 1. Introduction

Gel is a solid, jelly-like material whose properties can range from weak and soft to tough and hard. According to the solvent they contain, gels can be categorized as organogel or hydrogel [1]. Traditional polymer gels form three-dimensional network structures by covalent cross-linking. However, low molecular weight gelators (LMWGs) can aggregate into well-defined nano/microstructures via non-covalent interactions between gelator–gelator and gelator–solvents [2,3]. In recent years, LMWG-responsive hydrogels have been developed due to the integration of more controllable properties and functions. Generally, LMWGs are formed by various supramolecular forces, such as π-π stacking, hydrogen bonding, hydrophobic forces, and electrostatic interaction [4,5]. Typical LMWGs include amino-acid derivatives, carbohydrates, and peptides.

Azobenzenes have been successfully introduced into supramolecular assemblies via reversible *trans–cis* photochemical isomerisation upon exposure to different wavelengths of light. For example, Hao designed and synthesized two kinds of azobenzene derivatives (ADPMA and ADPSA), whose pH and other effective behaviors and gel-forming ability were investigated in detail [6]. Saito et al. incorporated the azobenzene group into photoresponsive surfactants, facilitating the assembly of low molecular weight gels with reversibly switchable viscoelasticities triggered by different stimuli [7]. Novel sugar-decorated nanofibers composed of azobenzene, and disaccharide lactones were synthesized by Ogawa et al. They focused on how to control these materials with light [8]. Our group also designed a novel azobenzene surfactant that exhibits facile, reversible, and dynamic gel–sol changes to stimuli (e.g., heat, pH, light). These characteristics are particularly suitable for applications [9,10,11]. Azobenzene compounds are known to interact with bacterial cell membranes. Their light-switching ability can modulate the membrane interactions of peptides; hence these compounds are used as light controllers in biological systems [12,13,14]. Photosensitive hydrogels hold tremendous promise for stem cell reprogramming, as they can control precise spatiotemporal over a wide range of parameters. At the same time, their attractive ability to be non-invasive has also attracted the interest of a large number of scientists [15,16,17]. The temperature-responsive and shear-thinning properties can be used as injectable hydrogels to encapsulate macromolecular, small-molecular, or cellular cargos in vivo.

The biological efficacy of compounds depends on the counter anions, the head groups, and the N-substituents on the quaternary ammonium moiety [18]. Azobenzene and quaternary ammonium compounds show various biocidal activities. In the quest for biologically active compounds, research includes isolation, characterization, and synthesis of novel compounds that can be used in medicine. Numerous publications describe the synthesis, structure-activity relationships of these compounds, such as germicidal, antibacterial, antifungal, and anticancer activities [19,20].

Therefore, in this paper, azophenyl symmetric amphiphiles with quaternary ammonium salt ends were synthesized, and their response behaviors and antibacterial activities were tested. Linking these functional groups into one molecule is expected to lead to the formation of novel gelling agents. This hydrogel will generate a thermal response which also shows a response to light irradiation. We found that functional units can be incorporated into the gel and that they retain their antimicrobial activity. Therefore, these responsive soft materials have many potential applications.

## 2. Results and Discussion

**Scheme 1:**
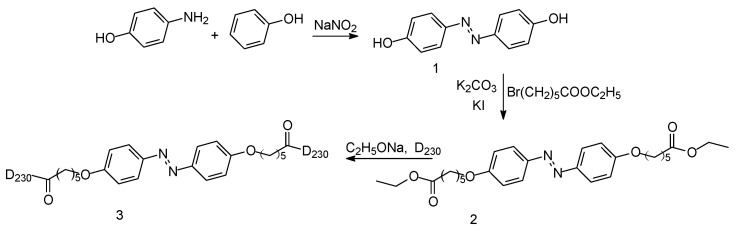
Synthesis of D-Azo-D_230_.

The synthesis and characterization of double azobenzene polyetheramine (D-Azo-D_230_) is described in the following information (Scheme 1).

### 2.1. ^1^H NMR Analysis

**Figure 1:**
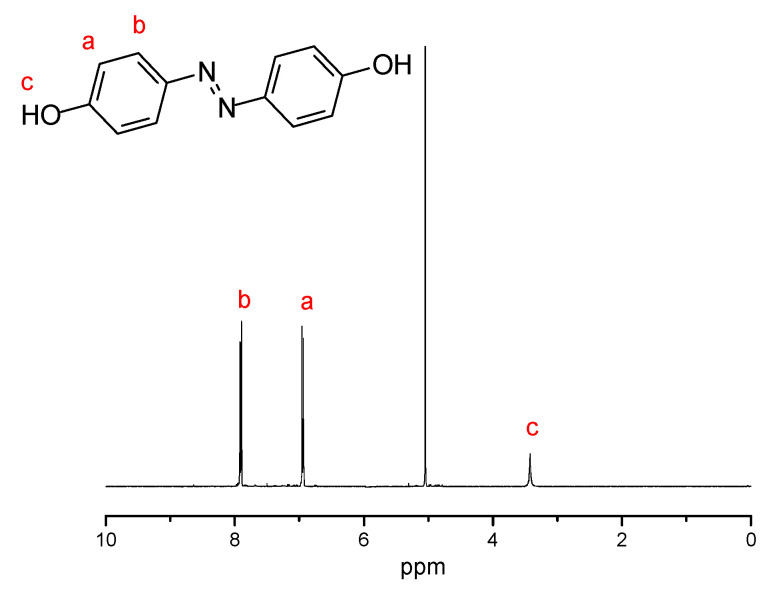
^1^H NMR spectrum of compound **1** in methanol-d4.

**Figure 2:**
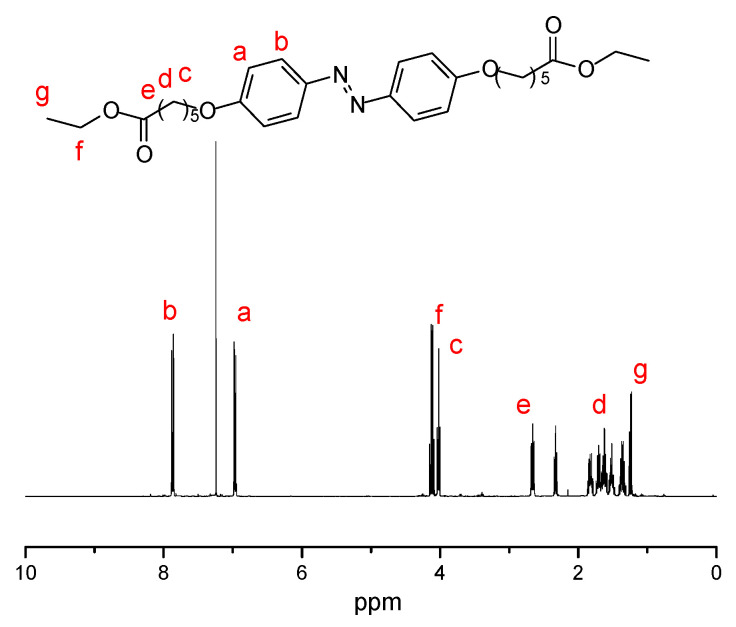
^1^H NMR spectrum of compound **2** in chloroform-d.

**Figure 3:**
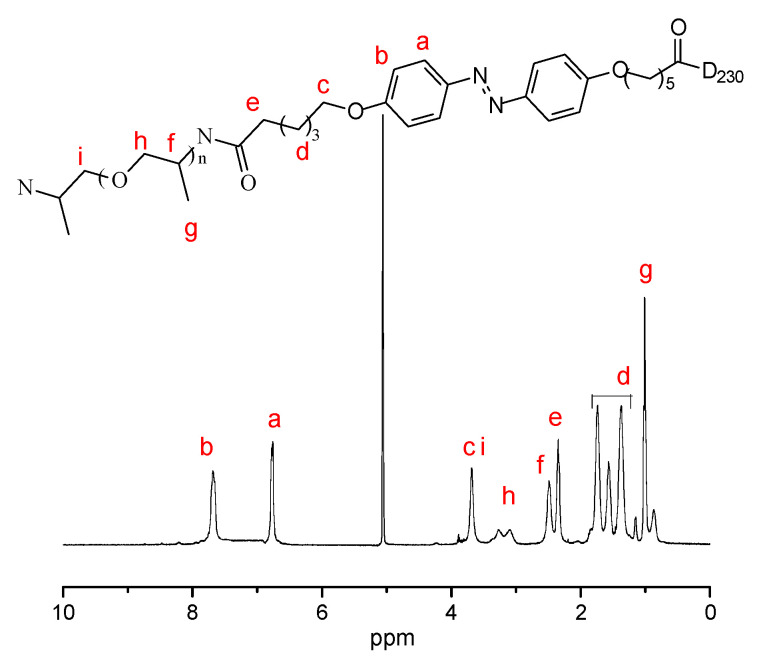
^1^H NMR spectrum of compound **3** in methanol-d4.

Figure 1, Figure 2 and Figure 3 present the ^1^H NMR spectrum of compounds **1–3**. Figure 1 shows the chemical structure of compound **1** and its characteristic peaks of different types of hydrogen. The chemical structure of compound **1** is simple, and only three types of hydrogen corresponded to the characteristic peaks. There are eight different kinds of hydrogen, and the different kinds correspond to their characteristic peaks one by one in Figure 2. Figure 3 is the chemical structure of D-Azo-D_230_ and its characteristic peaks. The peak observed at 2.2 ppm belongs to the carbonyl group, and the signals of 1.1–1.8 ppm match the methylene groups; a peak at 1.0 ppm was observed that might belong to the –CH_3_ present on the structure of D_230_. A new broad peak appeared at 2.5–3.5 ppm corresponding to the formation of amide groups. The main ^1^H NMR peaks corresponding to the hydrogens of compounds **1–3** have been marked in the Figure 1, Figure 2 and Figure 3.

### 2.2. Gelation Ability

**Figure 4:**
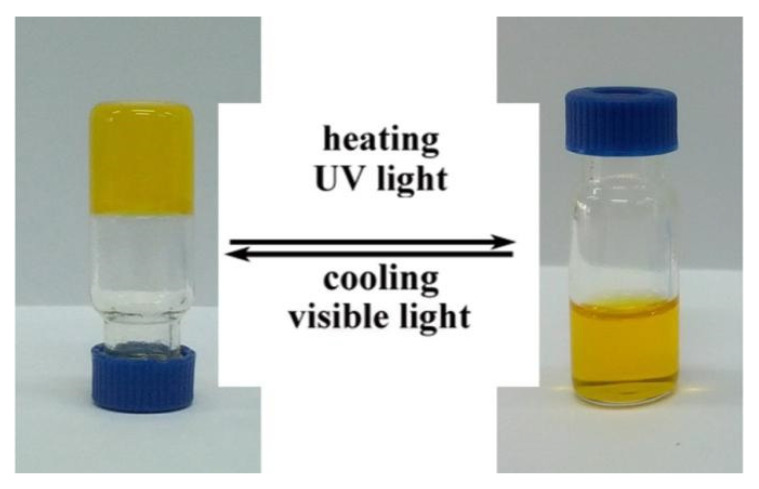
Photographs of the D-Azo-D_230_ in water 5.0 wt%: 20 °C (**left**); 80 °C (**right**).

**Figure 5:**
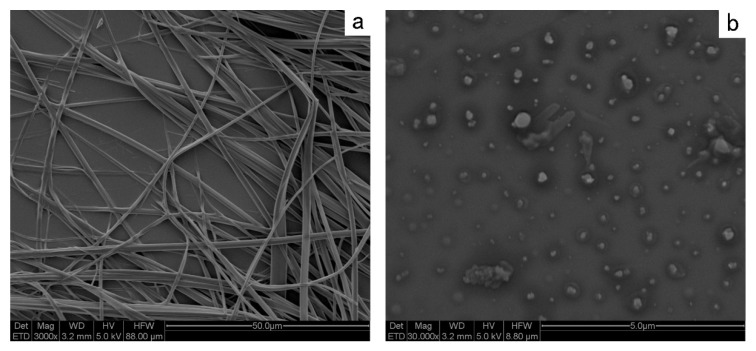
SEM of the D-Azo-D_230_ fibers (**a**) and residual solution of the hydrogel (**b**).

The gelation ability was measured in water (Figure 4). In general, the solution (D-Azo-D_230_) was heated until a solution was obtained (80 °C). Then it was allowed to return to room temperature. The gel formation ability was tested by inversion of the vials. In 5.0 wt%, the yellow-colored hydrogel could be generated rapidly within 30 min. After inverting the vial for three hours, the hydrogel could still stably exist on the bottom of the vial without slipping off, which showed the stability of the hydrogel. For the in situ observation of the fibrous structure, the hydrogel was put into liquid nitrogen. Then the product was lyophilized. Three-dimensional entangled supramolecular fibers can clearly be seen under SEM (Figure 5). The SEM revealed that D-Azo-D_230_ formed three-dimensional entangled supramolecular fibers. The structures and modes of formation of the self-assembled fibrillar networks (SAFINs) were similar to our groups’ previous works [10]. Similarly, under irradiating of UV light, the fibers were unstable, and the solution was formed (Figure 4). The nature and size of the fiber was dependent on the structure of the gelator. The gelator showed fibrous morphology with an average fiber thickness of 600–800 nm, and the length of the fibers was found to be several millimeters. Some of the fibers were associated with each other to form width fibers of approximately 2 μm. It should be noted that the gelator itself need not all be present in the nanofibers. A certain percentage of the gelator is generally present in solution. Figure 5b shows that the gelator state was transformed into vesicular structures.

### 2.3. Thermo Response

**Figure 6:**
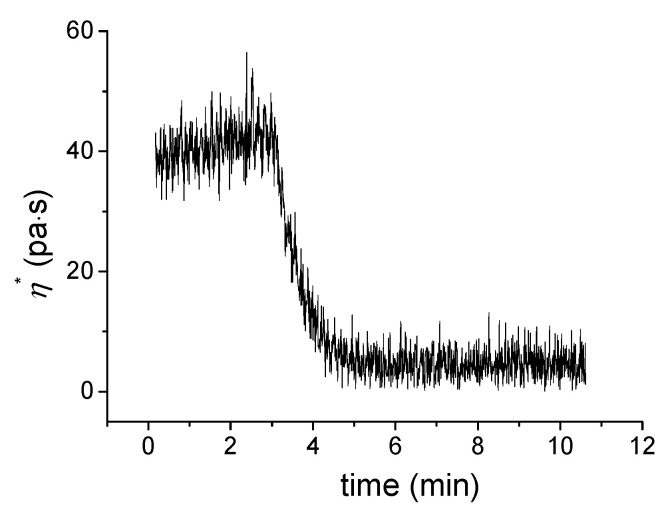
Plot of viscosity against the temperature of D-Azo-D_230_ in water (5.0 wt%). The data were obtained at a constant frequency of 5 rad/s and a strain of 1.0%.

The reversible nature of the non-covalent interactions of hydrogel could respond to thermo stimuli. Photographs of the gel–sol sample procedure are shown in Figure 4. A yellow colored solution in water (5 wt%) was formed by heating. A rheological study showed the change in viscosity when temperature changes (Figure 6). The initial temperature was 10 °C. After keeping the temperature for 2 min, the temperature was increased at a rate of 10 °C/min. Finally, the temperature was maintained at 70 °C. At 10 °C the sample’s viscosity was about 45 Pa·s. Following the increase in temperature to 20 ℃, a dramatic decrease of viscosity was observed. Viscosity reached a new steady state of 3 Pa·s at 50 °C. This indicates that the hydrogel was completely converted into an aqueous solution. Upon cooling, the solution could regenerate to hydrogel. However, the speed of gel formation has a close relationship with the concentration of the gelling factor. The gel formed rapidly within 60 min at 15 wt% but slower, more than 12 h, at 5.0 wt%. This reversible gel–sol phase transition could be repeated many times.

### 2.4. Light Response

**Figure 7:**
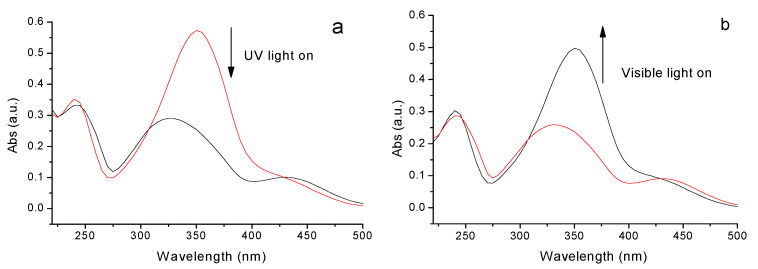
D-Azo-D_230_ in water at 20 °C (0.002 wt%): (**a**) change in absorption of UV-Vis spectra over time under UV light (365 nm, 3.8 mW/cm^2^); (**b**) change in absorption of UV-Vis spectra over time under visible light (12.1 mW/cm^2^).

The azobenzene compound was able to switch between *trans* and *cis* forms under alternating UV and visible light irradiation [21]. D-Azo-D_230_ showed its reversible photoisomerization, and the UV-Vis spectrum was used to study the reversible photoisomerization under UV and visible light. *Trans* in a water solution showed a strong absorption band centered at around 360 nm due to the *trans* form of the azobenzene group. This kind of absorbance corresponded to π-π* and n-π* electronic transition, respectively. The irradiation of UV light at 365 nm was accompanied by the decay of the maximum absorption band at around 360 nm and the generation of a new absorption at 440 nm. In addition, the absorbance at 360 nm decreased from 0.58 to 0.27 which indicated the trans-cis isomerization almost wholly completed. This photo-induced isomerization was reversible (Figure 7b), as the D-Azo-D_230_ in the *cis* form relaxes to the *trans* form within a few minutes after exposure to visible light or slower in the dark which is important for practical applications.

### 2.5. Antimicrobial Activity

**Figure 8:**
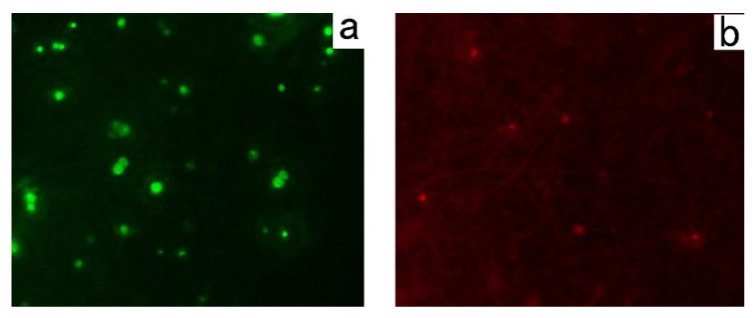
Human fibroblasts L929 supportive properties of D-Azo-D_230_ hydrogels. After hydrogel swelling in culture medium for 24 h, L929 were seeded onto the hydrogels surface and incubated for 24 h: (**a**) live cells on the hydrogel; (**b**) dead cells on the hydrogel.

**Figure 9:**
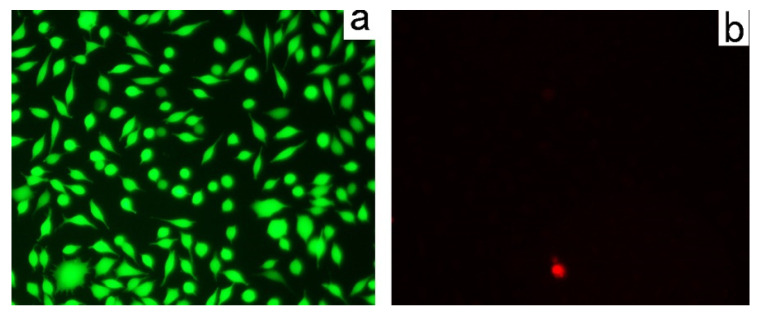
Human fibroblasts L929 in control well stained by live and death assay: (**a**) confocal images of L929; (**b**) dead cells in control well.

Hydrogels have experienced rapid growth in recent decades as they play an invaluable role in medicine by restoring function and promoting healing in people after disease or injury [22]. To verify the biocompatibility of D-Azo-D_230_ hydrogels, live and death assays were used for in vitro cell viability studies of the supportive properties of human fibroblasts L929. Figure 8 shows the surviving cells of D-Azo-D_230_ hydrogel after 1 day of culture. The free-standing samples were used as substrates to study the morphology and viability of the cells cultivated on the hydrogel surfaces. Compared with those in control wells (Figure 9a,b), the density of human fibroblasts L929 on the surface of the hydrogel was lower than the cells per cm^2^ due to the highly assembled D-Azo-D_230_ backbone, demonstrating that the gel aggregated into fibers suitable for cell growth. Additionally, the gelator itself need not all be present in the network-forming assemblies (gel fraction); a certain percentage of the gelator is generally present in solution (sol fraction). Thus, there is the possibility of gelator exchange occurring between the gel and sol states [21]. However, molecular gelators on the solvent medium were easily swallowed by the cells, resulting in cytotoxicity and preventing cells from dividing and growing. The small molecule azobenzene gelator dissolved in the solution has an antibacterial effect and was not suitable for cell growth.

**Figure 10:**
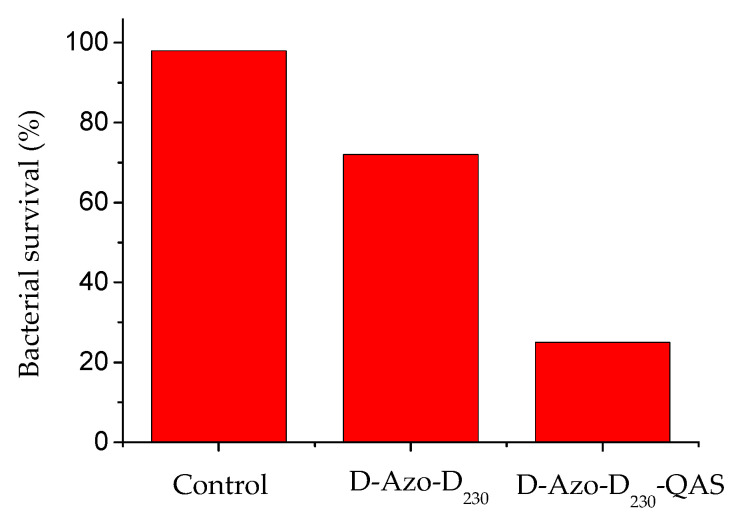
Antimicrobial activity of hydrogels tested against *E. coli*.

The capability of D-Azo-D_230_ to kill the bacteria was poor (Figure 8b) for it failed to kill the attached bacteria [23]. However, both azobenzene and the quaternary ammonium compound showed the different biocidal activities. In the quest for a biologically active form of this compound, the tertiary amine head groups of D-Azo-D_230_ were substituted for the quaternary ammonium moiety (quaternary ammonium salt of double azobenzene polyetheramine, D-Azo-D_230_-QAS) by using hydrochloric acid. The D-Azo-D_230_ and D-Azo-D_230_-QAS were tested against bacterial solutions of *E. coli*. The D-Azo-D_230_-QAS sample showed excellent inhibitory activity against *E. coli*. The whole bacterial population of that strain was almost eliminated in the D-Azo-D_230_-QAS sample (Figure 10). D-Azo-D_230_ reduced close to 25% of the bacteria, while D-Azo-D_230_-QAS eradicated 76% of the bacteria. These bacteria showed major resistance to D-Azo-D_230_ because it can self-assemble in aggregation in solution. This large volume of compound limits the diffusion of external agents into the cell. D-Azo-D_230_-QAS has a complete solubility and that makes it more susceptible to bacteria. The amount of previously loaded quaternary ammonium had a great influence on antimicrobial efficacy. All the above statistics support the idea that D-Azo-D_230_-QAS might have antimicrobial properties.

## 3. Conclusions

A photo-responsive azobenzene-based hybrid material was synthesized by a simple process. Its self-assembly behavior as well as solution and gel state were investigated. The presence of the azobenzene moiety allowed it to activate an efficient light-induced smart material, with light illumination reversing the *trans-cis* transition. *Live–dead assay* results showed that the hydrogels offer a hard approach for cell culture. However, compared to a conventional stimuli-responsive hydrogel showing sol–gel changes, this azobenzene compound and its quaternary ammonium compound show different biological activity against *E. coli.* bacteria. The biocidal activity of the quaternary ammonium compound is greater than that of its parent azobenzene compound. The photo-responsive antibacterial material presented in this research will provide new insights into fabrication of novel stimuli-responsive smart biomedical soft matter.

## 4. Materials and Methods

### 4.1. Materials and Methods

D_230_ was supplied by Huntsman Corporation, USA. Sodium nitrite was purchased from Sigma-Aldrich. All other chemicals were purchased from China National Pharmaceutical Group Co., Ltd. (Shanghai, China) and used without further purification. ^1^H NMR spectroscopy was tested on a 400 MHz Bruker Spectrometer (Bruker, Germany). Absorption of UV-Vis spectra was measured on a Cary 50-Bio UV-vis spectrophotometer in a quartz cuvette (Varian). Isomerisation was tested on an EXFO Acticure 4000 light source (working wavelength: 365 nm). The visible light intensity (12.1 mW/cm^2^). UV light intensity (3.8 mW/cm^2^). SEM was performed on a FEI Quanta 400F ESEM (FEI Co., Ltd., USA). The samples were examined at 5.0 kV accelerating voltage. The surfaces were observed using an Axio Observer A1 fluorescence microscope (Carl Zeiss Inc., Germany). Rheology was conducted on a rheometer with a temperature controller to maintain the temperature (TA Instruments, USA).

### 4.2. Synthesis of D-Azo-D_230_ and D-Azo-D_230_-QAS

Compound **1**: A solution of sodium nitrite (1 M, 15 mL) at 0 °C, was added drop-wise to 4.3 g (26.70 mmol) of aniline in a mixture of 10 mL HCl and 30 mL of water. After stirring for 30 min, the diazonium salt was formed. Then, 3.35 g of phenol (35 mmol) was added. The reaction was stirred for 1 h. The solution was made basic with 100 mL of a saturated solution of NaHCO_3_. After 30 min, the reaction solution was acidified by adding acetic acid or HCl (2 M). Then the formed precipitate was filtered and washed by water. The product was isolated by recrystallization with ethanol.

Compound **2**: A total of 18.5 mmol of compound **1**, KI (0.200 g) and K_2_CO_3_ (7.7 g, 55.0 mmol) was dissolved in acetone (100 mL), under a nitrogen atmosphere. After 10 min stirring, 45 mmol of ethyl 6-bromohexanoate was added drop wise via syringe. Then the solution was heated to reflux. The mixture was stirred overnight. After cooling down to room temperature, the salt solid was filtered off, and acetone was evaporated. The product was recrystallized with ethanol.

D-Azo-D_230_: 6.0 g D_230_ dissolved in 10 mL dichloromethane and 0.1 g sodium ethylate was added in the flask at −10 °C. The corresponding compound **2** (1.0 g) dissolved in dichloromethane (15 mL) was added within 30 min. Then the reaction mixture was heated to 30 °C and stirred overnight. The solid was formed and filtered off. The filter was washed by dichloromethane. Finally, the filtrate was collected and concentrated. The product was dried in the vacuum oven at 30 °C overnight.

D-Azo-D_230_-QAS: 1 g D-Azo-D_230_ dissolved in 10 mL water at 60 °C, 5 mL HCl (1 M) was added slowly. After stirring for 60 min, the quaternary ammonium salt was formed. Then the solution was concentrated and recrystallized. The solid was dried in the vacuum oven at 50 °C overnight.

### 4.3. Biocompatibility of the Hydrogels

We cultivated human fibroblasts L929 both on the surfaces of the hydrogels and in bulk free-standing samples to have a direct impression on the biocompatibility of the hydrogels. Green-labeled cells represent viable cells, while red-labeled cells indicate dead cells. The detail of the experiment was prepared with a similar method in the literature [24].

### 4.4. Antimicrobial Activity Pretreatments

Escherichia coli (*E. coli*) was chosen as a model bacterium for bacteria-related experiments. Prior to tests, the bacteria were cultured in the exponential phase through overnight incubation at 37 °C. After the incubation step, each treatment medium (100 µL) was taken and passed through a 96-well plate. The absorbance was measured at 600 nm using a microplate reader to determine the percentage of bacterial survival [23].

## Data Availability

Not applicable.
